# Supplementary material for: Objective and Perceived Neighborhood Greenness of Students Differ in Their Agreement in Home and Study Environments
Source: Int J Environ Res Public Health. 2020 May 14;17(10):3427. doi: 10.3390/ijerph17103427 (PMC7277367; doi:10.3390/ijerph17103427)
Supplement: Supplementary file 1 [file ijerph-17-03427-s001.pdf]

Table S1. Frequency distribution of the quintiles from objective to perceived measures.

**Greenness at Home**							
		**Objective Greenness Quintiles**					
		**1**	2	3	4	5	Missing
Perceived Greenness Quintiles	1	25	14	11	11	8	7
	2	10	19	10	16	12	10
	3	11	11	15	13	13	13
	4	12	14	17	13	12	24
	5	3	4	6	9	16	20
	Missing	0	0	0	0	0	0
		Objective Greenness Quintiles					
		1	2	3	4	5	Missing
Perceived Presence of Greenness Quintiles	1	29	19	14	9	7	8
	2	5	10	3	11	8	4
	3	16	20	26	21	19	22
	4	5	6	7	8	3	9
	5	7	7	9	11	24	31
	Missing	0	0	0	0	0	0
**Greenness at University**							
		Objective Greenness Quintiles					
		1	2	3	4	5	Missing
Perceived Greenness Quintiles	1	12	12	10	10	19	2
	2	20	18	18	24	15	3
	3	16	17	21	16	15	1
	4	12	11	11	12	8	1
	5	10	17	17	12	13	3
	Missing	1	0	0	0	0	0
		Objective Greenness Quintiles					
		1	2	3	4	5	Missing
Perceived Presence of Greenness Quintiles	1	12	13	6	10	18	1
	2	21	12	26	19	18	4
	3	10	9	11	16	7	0
	4	10	22	11	10	14	3
	5	16	19	23	18	13	2
	Missing	2	0	0	1	0	0

Table S2. Collinearity statistic.

Variable	Tolerance	Variance Inflation Factor
Estimation of Greenness at Home		
Objective Greenness at Home	0.90	1.11
Objective Greenness at University	0.83	1.08
Perceived Greenness at Home	0.82	1.22
Perceived Greenness at University	0.82	1.23
Gender	0.94	1.06
Marital Status	0.84	1.19
Age	0.57	1.77
Income	0.67	1.49
Education	0.81	1.24
Estimation of Greenness at University		
Objective Greenness at Home	0.90	1.11
Objective Greenness at University	0.93	1.08
Perceived Greenness at Home	0.82	1.22
Perceived Greenness at University	0.82	1.23
Gender	0.94	1.06

Marital Status	0.84	1.19
Age	0.57	1.77
Income	0.67	1.49
Education	0.81	1.24
Estimation of Greenness at Home		
Objective Greenness at Home	0.91	1.11
Objective Greenness at University	0.92	1.08
Perceived Presence of Greenness at Home	0.82	1.22
Perceived Presence of Greenness at University	0.84	1.19
Gender	0.94	1.06
Marital Status	0.84	1.19
Age	0.57	1.77
Income	0.67	1.49
Education	0.81	1.24
Estimation of Greenness at University		
Objective Greenness at Home	.91	1.11
Objective Greenness at University	0.92	1.08
Perceived Presence of Greenness at Home	0.82	1.22
Perceived Presence of Greenness at University	0.84	1.19
Gender	0.94	1.96
Marital Status	0.84	1.19
Age	0.57	1.77
Income	0.67	1.49
Education	0.81	1.24

Table S3. Correlation matrix.

	OG home	OG university	PG home	PG university	Perceived Presence Home	Perceived Presence University	Gender	Marital status	Age	Income	Education
OG home											
OG university	*r* = -0.05<br>*p* = 0.250										
PG Home	*r* = 0.18<br>*p* = 0.004	*r* = -0.10<br>*p* = 0.083									
PG University	*r* = -0.19<br>*p* = 0.003	*r* = -0.10<br>*p* = 0.073	*r* = 0.32<br>*p* < 0.001								
P. Presence Home	*r* = 0.20<br>*p* = 0.001	*r* = -0.11<br>*p* = 0.050	*r* = 0.96<br>*p* < 0.001	*r* = 0.31<br>*p* < 0.001							
P. Presence University	*r* = -0.15<br>*p* = 0.015	*r* = -.09<br>*p* = 0.096	*r* = 0.31<br>*p* < 0.001	*r* = 0.96<br>*p* < 0.001	*r* = 0.31<br>*p* < 0.001						
Gender	*r* = 0.02<br>*p* = 0.390	*r* = 0.07<br>*p* = 0.161	*r* = -0.003<br>*p* = 0.482	*r* = -0.02<br>*p* = 0.373	*r* = 0.02<br>*p* = 0.363	*r* = 0.02<br>*p* = 0.388					
Marital status	*r* = -0.02<br>*p* = 0.375	*r* = -0.08<br>*p* = 0.117	*r* = 0.04<br>*p* = 0.285	*r* = 0.07<br>*p* = 0.159	*r* = 0.02<br>*p* = 0.390	*r* = 0.07<br>*p* = 0.169	*r* = 0.12<br>*p* = 0.038				
Age	*r* = 0.03<br>*p* = 0.357	*r* = 0.23<br>*p* < 0.001	*r* = 0.08<br>*p* = 0.131	*r* = -0.05<br>*p* = 0.239	*r* = 0.09<br>*p* = 0.106	*r* = -0.01<br>*p* = 0.445	*r* = 0.14<br>*p* = 0.022	*r* = -0.29<br>*p* < 0.001			
Income	*r* = 0.01<br>*p* = 0.427	*r* = 0.14<br>*p* = 0.018	*r* = 0.01<br>*p* = 0.449	*r* = 0.02<br>*p* = 0.407	*r* = 0.01<br>*p* = 0.455	*r* = .02<br>*p* = 0.410	*r* = 0.08<br>*p* = 0.117	*r* = -0.32<br>*p* < 0.001	*r* = 0.54<br>*p* < 0.001		
Education	*r* = 0.06<br>*p* = 0.214	*r* = 0.06<br>*p* = 0.191	*r* = 0.06<br>*p* = 0.208	*r* = -0.08<br>*p* = 0.114	*r* = 0.06<br>*p* = 0.205	*r* = -0.08<br>*p* = 0.136	*r* = -0.04<br>*p* = 0.290	*r* = 0.44<br>*p* = 0.001	*r* = 0.41<br>*p* < 0.001	*r* = 0.22<br>*p* = 0.001	

Table S4. ANOVA results of the comparison of objective and perceived greenness with relevant outcomes.

	F	df	p	Partial η^2
Mental Health				
Estimates at home	0.34	2, 284	.710	.002
Estimates at university	4.06	2, 284	.018	.03
Estimates at home and at university	0.96	4, 284	.430	.01
Physical Activity in MET-h				
Estimates at home	0.02	2, 250	.986	< .001
Estimates at university	0.49	2, 250	.611	.004
Estimates at home and at university	0.25	4, 250	.907	.004
Sedentariness in hours spent sitting per day				
Estimates at home	2.92	2, 282	.056	.02
Estimates at university	0.74	2, 282	.478	.01
Estimates at home and at university	1.05	4, 282	.380	.02
BMI				
Estimates at home	0.80	2, 280	.450	.01
Estimates at university	1.40	2, 280	.249	.01
Estimates at home and at university	0.52	4, 280	.720	.01

Table S5. ANOVA results of objective and perceived greenness dependent on gender and marital status.

	F	***df***	***p***	**Partial η^2**
Greenness at Home – Within Measures				
Greenness	420.38	1, 271	< 0.001	0.61
Greenness x Marital status	0.52	2, 271	0.593	0.004
Greenness x Gender	0.34	1, 271	0.562	0.001
Greenness x Marital status x Gender	0.19	2, 271	0.824	0.001
Greenness at Home – Between Measures				
Marital status	0.54	2, 271	0.582	0.004
Gender	0.14	1, 271	0.707	0.001
Marital status x Gender	0.72	2, 271	0.489	0.01
Greenness at University – Within Measures				
Greenness	315.55	1, 328	< 0.001	0.49
Greenness x Marital status	0.71	2, 328	0.493	0.004
Greenness x Gender	0.01	1, 328	0.933	< 0.001
Greenness x Marital status x Gender	0.35	2, 328	0.702	0.002
Greenness at University – Between Measures				
Marital status	0.25	2, 328	0.780	0.002
Gender	0.81	1, 328	0.368	0.002
Marital status x Gender	0.53	2, 328	0.592	0.003
